# Supplementary material for: Identification of photoperiod-induced specific miRNAs in the adrenal glands of Sunite sheep (Ovis aries)
Source: Front Vet Sci. 2022 Jul 22;9:888207. doi: 10.3389/fvets.2022.888207 (PMC9354845; doi:10.3389/fvets.2022.888207)
Supplement: Supplementary file 1 [file Data_Sheet_1.ZIP › Supplementary file/miRDeep2 file.zip!/miRDeep2 file/Novel_Structure/SPLP42A3/Novel_335.pdf]

Provisional ID	:	NC_019484.2_38673
Score total	:	1.8
Score for star read(s)	:	-1.3
Score for read counts	:	0
Score for mfe	:	1.5
Score for randfold	:	1.6
Score for cons. seed	:	
Total read count	:	709
Mature read count	:	709
Loop read count	:	0
Star read count	:	0

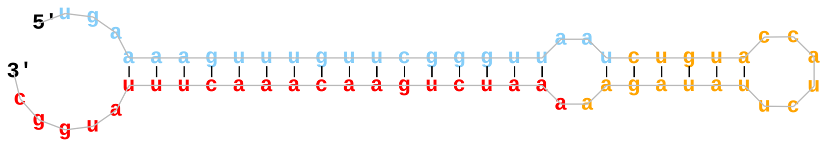

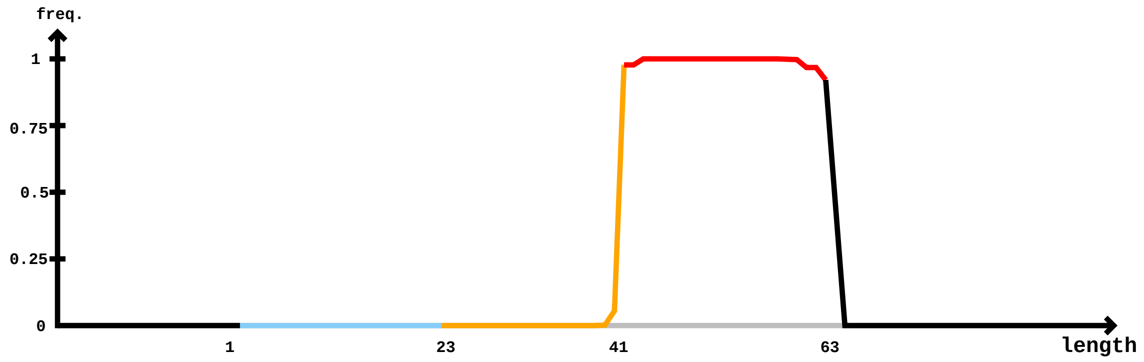

Star Mature

5'- caauccuagguguuggcugaaaaguuuguucggguuaaucuguaccaucuuauagaaaaaucugaacaaacuuuauggcuaacccaguauuuucucuucuuaccugaa -3'	exp		
........((.((((((((.((((((((((((((((..((((((......))))))..)))))))))))))))).))))))))))).......................	reads	mm	sample
....aaaaaCcugaacaaacuuua.................................	1	1	seq
....aaaaucugaacaaacuuu..................................	1	0	seq
....aaaaucugaacaaacuuuUu................................	18	1	seq
....aaaaucugaacaaacuuuUugg..............................	19	1	seq
....aaaucugaacaaacuuuUu................................	3	1	seq
....aaaucugaacaaacuuuUugg..............................	14	1	seq
....aaaucugaacaaacuuuUuggc.............................	637	1	seq
....aucugaacaaacuuuUuggc.............................	16	1	seq
